# Domestication is adaptive evolution in response to human use

**DOI:** 10.1073/pnas.2518220122

**Published:** 2025-12-15

**Authors:** Patrick L. Kohl

**Affiliations:** ^a^Department of Livestock Population Genomics, University of Hohenheim, Stuttgart 70599, Germany; ^b^Center for Biodiversity and Integrative Taxonomy, University of Hohenheim and Stuttgart State Museum of Natural History, Stuttgart 70599, Germany

According to a new definition of domestication, bed bugs and house sparrows are domesticated, whereas racing horses and broiler chickens are not. Lord et al. (1) suggest that domesticated populations are reliably distinguished from nondomesticated populations solely based on their current relationships with humans. Specifically, populations should be considered domesticated if they are obligately synanthropic (i.e., only exist within an anthropogenic niche) and self-sustaining.

While this radically new view seems liberating, it contradicts the basic consensus that domestication is a special case of evolution. In fact, according to their view, populations could switch from “human-tolerant” to “obligate synanthrope” and thus become “domestic” solely based on environmental, rather than evolutionary, change. As Lord et al. review, house cats (*Felis catus*) are obligate synanthropes in Europe. However, they thrive as wild populations in natural habitats when introduced to Australia, where they have no competitors and find abundant prey. Factual switches along the (ecological) synanthrope spectrum can also be realized by alterations of the original habitat. For example, populations that tolerate human disturbance can become obligate synanthropes simply because humans destroy natural landscapes (consider cavity-nesting species that depend on artificial nest boxes after the loss of pristine forests). Lord et al. contradict themselves when appreciating the possibility of “domestication through […] changes in environment,” while at the same time implying that domestic populations depend on their anthropogenic niches for evolutionary reasons.

Furthermore, Lord et al.’s requirement that populations must be “self-sustaining” to be considered domestic is arbitrary. Where is the line between an anthropogenic niche with acceptable human-mediated input of resources and a laboratory or animal production environment? Why does it make a difference whether blood-sucking human parasites live in human structures (bed bugs: beds, body lice: clothes, both considered domesticated by Lord et al.) or on human bodies entirely (head lice, not considered domesticated)?

Lord et al. debunk the assumption that human intentionality is key for domestication, and they convincingly argue against overloading the domestication concept by including criteria of mutualism and coevolution. However, by considering obligate synanthropy as an evolutionary outcome, they make an error analogous to the much-criticized conflation of current utility with historical origins.

Despite all valid criticism of previous interpretations of domestication, it is still worth considering the very core of the original, Darwinian concept of domestication before introducing a new definition. In essence, populations are classically considered under domestication if they provide a service/resource to humans and evolve toward higher rates of service/resource provision in their human-modified environment [modified after (2)]. The characteristic result is adaptive genetic change that facilitates human use, regardless of whether this is due to artificial (i.e., conscious breeding) or natural selection, or how the interspecific relationship unfolded historically (3). Whether a domestication process is at work can be investigated by testing for adaptive heritable differences among managed and wild populations of the same species (e.g., ref. 4), or between extant managed populations and their presumed wild progenitors (5, 6). Such comparisons are not always possible; however, this is a general problem in evolutionary biology.

## References

[1] K. A. Lord, G. Larson, R. G. Allaby, E. K. Karlsson, A universally applicable definition for domestication. Proc. Natl. Acad. Sci. U.S.A. **122**, e2413207122 (2025). 40372471 10.1073/pnas.2413207122 PMC12146738

[2] M. D. Purugganan, What is domestication? Trends Ecol. Evol. **37**, 663–671 (2022). 35534288 10.1016/j.tree.2022.04.006

[3] G. Larson, D. Q. Fuller, The evolution of animal domestication. Annu. Rev. Ecol. Evol. Syst. **45**, 115–136 (2014).

[4] C. E. Stanley, R. J. Kulathinal, Genomic signatures of domestication on neurogenetic genes in *Drosophila melanogaster*. BMC Evol. Biol. **16**, 6 (2016). 26728183 10.1186/s12862-015-0580-1 PMC4700609

[5] L. A. F. Frantz, D. G. Bradley, G. Larson, L. Orlando, Animal domestication in the era of ancient genomics. Nat. Rev. Genet. **21**, 449–460 (2020). 32265525 10.1038/s41576-020-0225-0

[6] M. Schubert , Prehistoric genomes reveal the genetic foundation and cost of horse domestication. Proc. Natl. Acad. Sci. U.S.A. **111**, E5661–E5669 (2014). 25512547 10.1073/pnas.1416991111 PMC4284583

